# Genome-wide SNP data reveal genetic relatedness and structure in an ex-situ population of threatened Fea's muntjac (*Muntiacus feae*) (Artiodactyla, Cervidae)

**DOI:** 10.3897/zookeys.1283.186911

**Published:** 2026-06-26

**Authors:** Pannawat Supapannachart, Sithichoke Tangphatsornruang, Wirulda Pootakham, Chutima Sonthirod, Sissades Tongsima, Pongsakorn Wangkumhang, Alisa Wilantho, Ampika Thongphakdee, Saowaphang Sanannu, Pongsatorn Kongjak, Thewarach Vechmanus, Supatsorn Panyalert, Gunnaporn Suriyaphol

**Affiliations:** 1 Biochemistry Unit, Department of Physiology, Faculty of Veterinary Science, Chulalongkorn University, Bangkok 10330, Thailand Department of National Parks, Wildlife and Plant Conservation Bangkok Thailand; 2 National Center for Genetic Engineering and Biotechnology (BIOTEC), National Science and Technology Development Agency, Pathum Thani 12120, Thailand Biochemistry Unit, Department of Physiology, Faculty of Veterinary Science, Chulalongkorn University Bangkok Thailand; 3 Animal Conservation and Research Institute, Zoological Park Organization of Thailand under the Royal Patronage of H.M. the King, Bangkok 10300, Thailand Center of Excellence in Wildlife Exotic and Aquatic Animal Pathology, Faculty of Veterinary Science, Chulalongkorn University Bangkok Thailand; 4 Chiang Mai Zoo, Zoological Park Organization of Thailand under the Royal Patronage of H.M. the King, Chiang Mai 50200, Thailand Animal Conservation and Research Institute, Zoological Park Organization of Thailand under the Royal Patronage of H.M. the King Bangkok Thailand; 5 Department of National Parks, Wildlife and Plant Conservation, Bangkok 10900, Thailand Chiang Mai Zoo, Zoological Park Organization of Thailand under the Royal Patronage of H.M. the King Chiang Mai Thailand; 6 Center of Excellence in Wildlife Exotic and Aquatic Animal Pathology, Faculty of Veterinary Science, Chulalongkorn University, Bangkok 10330, Thailand National Center for Genetic Engineering and Biotechnology (BIOTEC), National Science and Technology Development Agency Pathum Thani Thailand

**Keywords:** Cervid, conservation genomics, deer, Fea's muntjac, genetic relatedness, hybridization, RADseq

## Abstract

Fea’s muntjac (*Muntiacus feae*) is a poorly known cervid restricted to forested regions along the Thai–Myanmar border, and its ex-situ population in Thailand has declined to critically low numbers. Limited founder size and incomplete pedigree records raise concerns about inbreeding and the loss of genetic diversity, underscoring the need for genome-based assessments to support effective conservation management. In this study, restriction site-associated DNA sequencing (RADseq) was applied to investigate genetic diversity, relatedness, and population structure among the remaining ex-situ Fea’s muntjac individuals in Thailand, including one putative hybrid. Genome-wide single-nucleotide polymorphisms (SNPs) were generated and analyzed using phylogenetic reconstruction, Bayesian clustering, multidimensional scaling (MDS), and identity-by-descent (IBD) approaches. Phylogenetic, ADMIXTURE, and MDS analyses consistently revealed clear genetic structuring corresponding to institution of origin, whereas the putative hybrid formed a distinct genetic lineage. IBD analyses identified both first- and second-degree relationships among individuals, largely corroborating available pedigree records, while also revealing previously undocumented relatedness, emphasizing the limitations of pedigree-based management in small ex-situ populations. Notably, one individual exhibited no detectable genetic relatedness to the others and consistently formed an independent lineage across analyses, indicating the presence of unique genetic variation with high conservation value. Collectively, these results demonstrate the utility of RADseq-derived genomic data for resolving kinship, validating pedigrees, and detecting hybridization in extremely small ex-situ populations. This study represents the first comprehensive genome-wide genetic assessment of ex-situ Fea’s muntjac in Thailand and establishes an essential genomic baseline to inform breeding decisions and support long-term conservation planning for this highly threatened cervid.

## Introduction

Muntiacine deer represent one of the most basal lineages within the family Cervidae. Their distinctive traits include deciduous antlers and markedly elongated upper canines, placing them evolutionarily between the antlerless, tusked cervid lineages of the Oligocene and the antlered, tuskless species that emerged during the early Pliocene (Dong 2007; Cabrera and Stankowich 2020). These characteristics highlight the importance of muntjacs for understanding cervid evolutionary history. Ecologically, muntjacs also play important roles as prey for medium- and large-sized predators and contribute to forest ecosystem dynamics through browsing and trampling activities (Crawley 1997).

Since 1996, Fea’s muntjac (*Muntiacus feae* (Thomas & Doria, 1889)) has been classified as “Data Deficient (DD)” on the IUCN Red List, reflecting persistent uncertainty regarding its population size, distribution, and conservation status (Timmins et al. 2016). In Thailand, the species is recognized as one of the 21 conserved species and is listed as Endangered (EN) under the Thailand Red List (Office of Natural Resources and Environmental Policy and Planning [ONEP] 2017; Office of the Council of State 2019). Fea’s muntjac is primarily restricted to forested habitats along the Thai–Myanmar border, with recent evidence extending its known distribution to southwestern China and Tibet (Wang et al. 2022). The species faces ongoing threats from habitat loss due to deforestation and hunting pressure, factors that likely contribute to population decline and increasing geographic and genetic isolation (Sabu et al. 2021). Alarmingly, the ex-situ population in Thailand has declined to fewer than three breeding pairs, underscoring its precarious conservation status (Office of the Council of State 2019). Such severe demographic constraints raise serious concerns regarding the erosion of genetic diversity and increased inbreeding risk, both of which may compromise long-term adaptability and population viability (Steinmetz et al. 2010). Consequently, a comprehensive assessment of the genetic composition of the remaining ex-situ individuals is crucial to support conservation planning and development of effective future management strategies (Allendorf et al. 2010; Frankham 2010).

Recent advances in genomic methodologies have enabled detailed investigations of population genetic structure in non-model and threatened species. Among these approaches, restriction site-associated DNA sequencing (RADseq) provides an efficient means of generating genome-wide single-nucleotide polymorphism (SNP) datasets without requiring complete genome coverage (Davey and Blaxter 2010). RADseq has been widely applied in conservation genomics studies of diverse taxa, including Eld’s deer (*Rucervus eldii* (McClelland, 1842)), forest musk deer (*Moschus berezovskii* Flerov, 1929), the Raso lark (*Alauda razae* Alexander, 1898), and the Asian king vulture (*Sarcogyps calvus* (Scopoli, 1786)), yielding critical insights into population structure, genetic diversity, and hybridization patterns (Fan et al. 2019; Dierickx et al. 2020; Pumpitakkul et al. 2023; Buthasane et al. 2024). Applying this framework to Fea’s muntjac offers an opportunity to clarify genetic relationships within the small ex-situ population in Thailand and to generate data directly relevant to conservation management, particularly breeding programs. Moreover, RADseq represents a cost-effective approach for conservation initiatives with limited financial resources compared with whole-genome resequencing.

The present study aimed to employ RADseq-derived SNP data to assess genetic diversity, relatedness, and population structure among the remaining ex-situ Fea’s muntjac individuals in Thailand. The resulting data are expected to provide essential baseline information to guide breeding decisions and strengthen long-term conservation strategies for this highly threatened cervid species.

## Material and methods

### Animal samples

**Figure 1.**
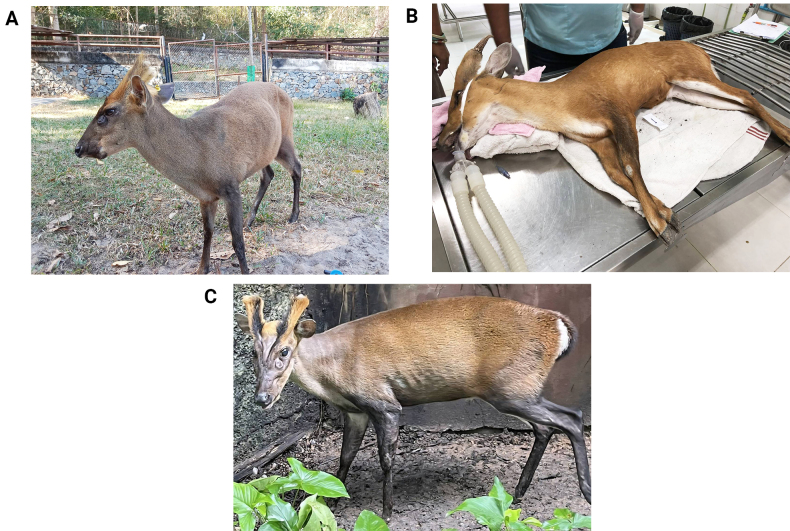
Three muntjac taxa maintained under the Zoological Park Organization of Thailand under the Royal Patronage of H.M. the King. **A**. Fea’s muntjac; **B**. Northern red muntjac; **C**. Putative Fea’s muntjac hybrid exhibiting intermediate morphological features between the two parental species.

**Figure 2.**
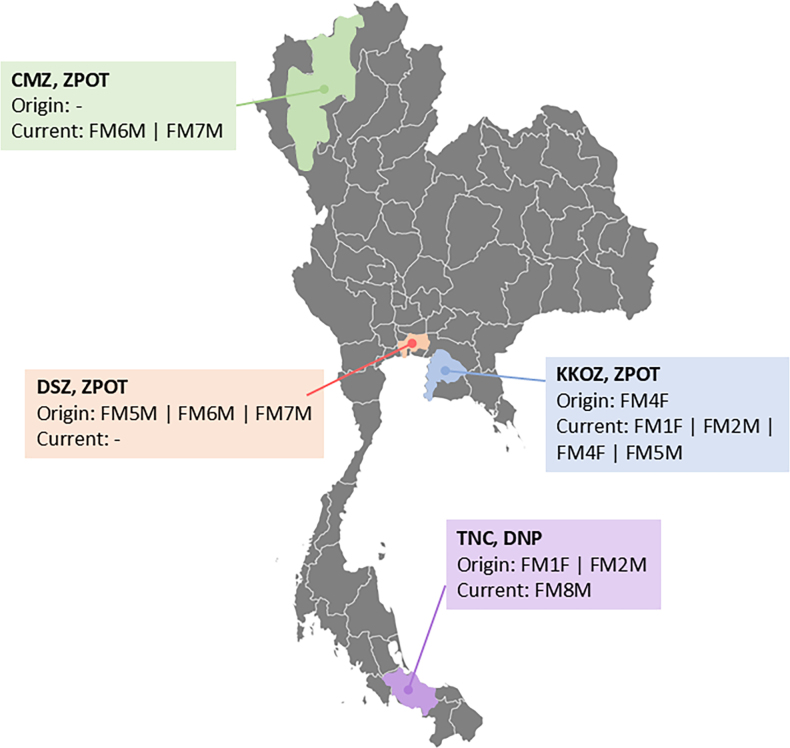
Sampling locations of the seven Fea’s muntjac individuals and one putative hybrid individual included in this study. “Origin” indicates the original holding institution of each individual, whereas “Current” indicates the facility where the individual was housed at the time of sample collection. Sampling sites: CMZ, Chiang Mai Zoo; DNP, Department of National Parks, Wildlife and Plant Conservation; DSZ, Dusit Zoo; KKOZ, Khao Kheow Open Zoo; TNC, Ton Nga Chang Wildlife Breeding Center; ZPOT, Zoological Park Organization of Thailand under the Royal Patronage of H.M. the King.

A total of seven Fea’s muntjac samples were included in this study. These comprised three whole-blood specimens collected from Khao Kheow Open Zoo (KKOZ) under the Zoological Park Organization of Thailand under the Royal Patronage of H.M. the King (ZPOT), including two individuals originating from Ton Nga Chang Wildlife Breeding Center (TNC) under the Department of National Parks, Wildlife and Plant Conservation (DNP) (FM1F and FM2M) and one individual originating from KKOZ (FM4F). In addition, two whole-blood specimens (FM6M and FM7M) were obtained from Chiang Mai Zoo (CMZ) under the ZPOT, one whole-blood specimen (FM8M) from TNC, and one archived muscle tissue specimen (FM5M) preserved in the blood and tissue bank of KKOZ were included in the study. Fea’s muntjac is a small-bodied cervid with an approximate body mass of 25 kg, characterized by a dark greyish-brown pelage, yellowish pigmentation on the forehead and at the bases of the ears, and distinct black facial stripes extending from the antler pedicles. In contrast, the northern red muntjac (*Muntiacus vaginalis* (Boddaert, 1785)) is slightly smaller, with an average body mass of approximately 20 kg and a uniformly reddish-brown coat, accompanied by similar black facial markings (Fig. 1). In addition, one whole-blood sample (HFM3M) was collected from a putative hybrid Fea’s muntjac at KKOZ. This individual exhibited mixed phenotypic traits, with the head, lower body and tail conforming to typical Fea’s muntjac morphology, whereas the dorsal coat showed a distinct reddish coloration characteristic of the northern red muntjac (Fig. 1). In addition to its intermediate coat coloration, HFM3M also exhibited atypical fecal characteristics, including pellet-form feces that appeared moister and more compressible than those typically observed in northern red muntjac, representing an intermediate phenotype. The current and original locations of all sampled individuals are summarized in Fig. 2, Table 1. Pedigree information for all animals was retrieved from institutional zoo records maintained by the ZPOT and used to infer documented parent–offspring and sibling relationships, thereby supporting the interpretation of genomic analyses. All sampling and laboratory procedures were conducted in accordance with protocols approved by the Chulalongkorn University Animal Care and Use Committee (CU-ACUC; approval no. 2131003).

**Table 1.** Sample information for Fea’s muntjac individuals included in this study. “Current” indicates the facility where each individual was housed at the time of sample collection, whereas “Origin” refers to the original holding institution of each individual.

**Sample I**D	**Sex**	**Location**		**Age (y)**	**Specimen**
		**Current**	**Origin**		
Fea’s muntjac (FM):					
FM1F	F	KKOZ	TNC	6	Whole blood
FM2M	M	KKOZ	TNC	6	Whole blood
FM4F	F	KKOZ	KKOZ	15	Whole blood
FM5M	M	KKOZ	DSZ	9	Muscle tissue
FM6M	M	CMZ	DSZ	14	Whole blood
FM7M	M	CMZ	DSZ	10	Whole blood
FM8M†	M	TNC	N/D	N/D	Whole blood
Putative hybrid Fea’s muntjac (HFM):					
HFM3M	M	KKOZ	N/D	6	Whole blood

†FM8M was excluded from downstream genomic analyses due to poor DNA quality and insufficient quantity. Abbreviations: DSZ, Dusit Zoo; KKOZ, Khao Kheow Open Zoo; TNC, Ton Nga Chang Wildlife Breeding Center; N/D, not determined. Age (y) indicates the age of each individual at the time of sample collection.

### DNA extraction, RAD sequencing and SNP identification

Genomic DNA was extracted from whole-blood samples of seven Fea’s muntjac individuals and the putative hybrid using the Quick-DNA Miniprep Plus Kit (Zymo Research, Irvine, CA, USA). Genomic DNA from the archived muscle tissue specimen was extracted separately using the Wizard HMW DNA Extraction Kit (Promega, Madison, WI, USA). DNA concentration and purity were assessed using a NanoDrop One spectrophotometer (Thermo Fisher Scientific, Waltham, MA, USA). Sample FM8M was excluded from further analyses due to poor DNA quality and insufficient quantity, likely resulting from blood clotting. The remaining samples were used for RAD library preparation and subsequently sequenced on the MGISEQ-2000RS platform (MGI Tech, Shenzhen, China). High-quality filtered reads were aligned to the Fea’s muntjac reference genome generated by our group (NCBI BioProject accession no. PRJNA931277). Single-nucleotide polymorphisms (SNPs) were identified using the Genome Analysis Toolkit (GATK) v. 4.2.3.0 (Van der Auwera and O’Connor 2020).

### Phylogenetic inference, genetic admixture and multidimensional scaling (MDS)

For phylogenetic analyses, individuals with more than 50% missing SNP genotypes were excluded. The remaining SNP dataset was filtered using VCFtools v. 0.1.16 to retain loci with a minor allele frequency (MAF) between 0.1 and 0.9, sequencing depth between 10× and 200×, and no missing genotype calls (Danecek et al. 2011). A maximum-likelihood phylogenetic tree was reconstructed using IQ-TREE v. 2.0.3 with 1000 bootstrap replicates (Minh et al. 2020). The MAF range of 0.1–0.9 was selected to retain well-genotyped informative variants while excluding rare variants prone to genotyping error and near-fixed variants with limited discriminatory power, consistent with filtering criteria applied in published RADseq studies of ex-situ endangered cervids using the same analytical pipeline (Pumpitakkul et al. 2023).

Population structure was further examined using multidimensional scaling (MDS) and Bayesian clustering analyses implemented in ADMIXTURE v. 1.3.0 (Alexander et al. 2009). Prior to these analyses, SNPs were pruned for linkage disequilibrium (LD) and subjected to quality control using PLINK v. 1.9. Variants with more than 10% missing genotypes, MAF < 0.05, significant deviation from Hardy–Weinberg equilibrium (p < 0.01), and individuals with ≥ 50% missing data were excluded. ADMIXTURE analyses were performed with K values ranging from 1 to 5, and the optimal number of clusters was determined by minimizing cross-validation error. All visualizations were generated using R v. 4.3.0. The MAF < 0.05 exclusion threshold follows the standard PLINK v. 1.9 quality-control pipeline commonly used for population structure inference (Purcell et al. 2007).

### Relatedness estimation using identity-by-descent (IBD)

Pairwise kinship relationships among the six Fea’s muntjac individuals and the putative hybrid were assessed using SNP data aligned to the Fea’s muntjac reference genome. Variant sites were filtered in VCFtools v. 0.1.16 using the following criteria: MAF between 0.1 and 0.9, read depth between 10× and 200×, retention of SNPs genotyped in all individuals, and < 50% missing data per individual (Danecek et al. 2011). The MAF lower bound of 0.10 for IBD estimation follows published recommendations for PLINK --genome analyses, which recommend a minimum MAF threshold of 5–10% to minimize bias from low-frequency variants in small or structured populations (Purcell et al. 2007; McMaster et al. 2025), and is consistent with filtering criteria applied in published RADseq studies of ex-situ endangered cervids (Pumpitakkul et al. 2023). Filtered SNPs were used to estimate identity-by-descent (IBD), using the “--genome” function in PLINK v. 1.9, which calculates pairwise PI_HAT values (Purcell et al. 2007). The resulting relatedness matrix was visualized as heatmaps in R v. 4.3.0, with additional formatting performed using an in-house Python script. Relatedness was visualized using a color gradient, with darker colors indicating closer relationships. PI_HAT values > 0.95 were interpreted as duplicate samples or identical twins; values ≥ 0.40 indicated first-degree relationships (e.g., parent-offspring or full siblings); values ≥ 0.20 indicated second-degree relationships (e.g., grandparents, grandchildren or half-siblings); values ≥ 0.10 indicated third-degree relationships (e.g., great-grandparents, great-grandchildren or first cousins); and values < 0.10 were considered unrelated (Purcell et al. 2007).

## Results

### Pedigree records

**Figure 3.**
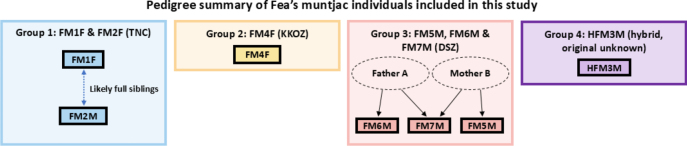
Pedigree relationships among six Fea’s muntjac (FM) individuals and one putative hybrid individual included in this study, reconstructed from available records. Brackets denote the animal’s original holding location.

Pedigree records identified four distinct groups among the studied Fea’s muntjac individuals analyzed in this study (Fig. 3). Group 1 comprised FM1F and FM2M, which are likely full siblings as records indicate a shared parental pair. Group 2 consisted solely of FM4F, the only individual originating from KKOZ, born in 2007, with no documented relatedness to individuals in the other groups. Group 3 included FM5M, FM6M and FM7M, all originating from Dusit Zoo (DSZ). Within this group, FM5M and FM7M shared the same mother, while FM6M and FM7M shared the same father, making FM7M both a maternal half-sibling of FM5M and a paternal half-sibling of FM6M. Group 4 comprised HFM3M, a putative hybrid individual lacking pedigree information, highlighting uncertainty regarding its ancestry relative to the other Fea’s muntjac groups.

### RADseq analysis and SNP calling

In total, 84 million high-quality paired-end reads, corresponding to 12.29 Gb of sequence data, were generated from the seven individuals. An average alignment rate of 97.13% was observed across all samples, corresponding to a mean of 11.57 million aligned reads per individual, when mapped to the Fea’s muntjac reference genome (NCBI BioProject accession no. PRJNA931277). Following variant calling, a total of 1,171,174 SNPs were identified across the Fea’s muntjac and hybrid samples and retained for downstream population genomic analyses (Suppl. material 1).

### RADseq-based population structure

**Figure 4.**
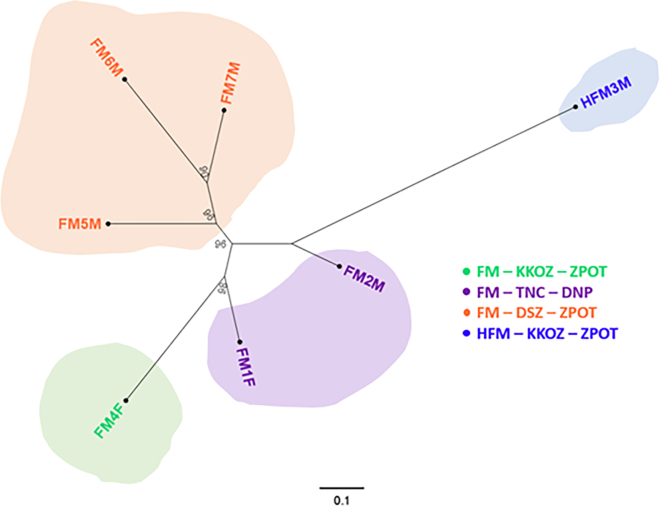
Maximum-likelihood phylogenetic tree of Fea’s muntjac individuals based on 9109 high-quality single-nucleotide polymorphisms (SNPs) with 1000 bootstrap replicates. The tree includes six Fea’s muntjac (FM) individuals and one putative hybrid Fea’s muntjac (HFM) and was constructed using the Fea’s muntjac reference genome. Numbers shown at the internal nodes (e.g., 86, 90, and 96) represent bootstrap support values (%) based on 1000 replicates. The institution of origin of each individual is indicated. The scale bar represents genetic distance.

After quality filtering, no individuals were excluded due to excessive missing data, resulting in a final dataset of 9,109 high-quality SNPs. These SNPs were used to infer population structure among ex-situ Fea’s muntjac by reconstructing a maximum-likelihood phylogenetic tree with 1000 bootstrap replicates under the VT+F+I substitution model. The resulting phylogenetic tree displayed distinct clustering patterns among individuals (Fig. 4). Within Fea’s muntjac, samples clustered largely according to their institution of origin. FM4F formed an independent lineage, whereas FM1F and FM2M clustered closely together. Individuals from DSZ (FM5M, FM6M, and FM7M) formed a well-supported cluster, consistent with their shared institution of origin. In contrast, the putative hybrid individual (HFM3M) occupied a clearly distinct branch, separated from all Fea’s muntjac samples.

**Figure 5.**
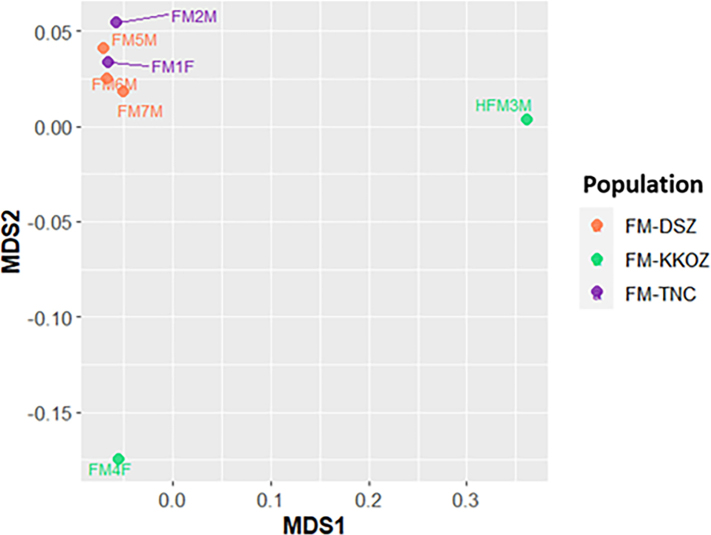
Multidimensional scaling (MDS) plot based on linkage disequilibrium (LD)-pruned SNPs, illustrating genetic relationships among six ex-situ Fea’s muntjac individuals and one putative hybrid individual. Points are colored by institution of origin.

To further assess ancestry patterns and genetic relationships, SNP data from six Fea’s muntjac individuals and the putative hybrid were filtered using PLINK v. 1.9. After initial filtering, 585,300 SNPs were retained and subsequently subjected to LD pruning, resulting in a final dataset of 67,209 SNPs for downstream analyses. MDS analysis revealed clear genetic separation among individuals from the three institutions of origin (Fig. 5). Individuals from TNC (FM1F and FM2M) clustered closely with the DSZ individuals (FM5M, FM6M, and FM7M), suggesting shared ancestry patterns. In contrast, FM4F formed a distinct lineage. The putative hybrid individual (HFM3M) was positioned separately from all Fea’s muntjac groups, consistent with its admixed genetic background. Overall, the MDS and phylogenetic analyses showed concordant clustering patterns, collectively supporting genetic differentiation among ex-situ populations and the distinct genetic status of the putative hybrid.

**Figure 6.**
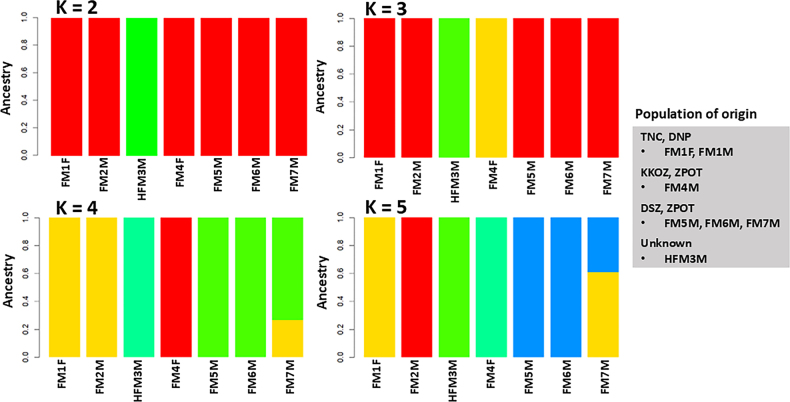
ADMIXTURE bar plots illustrating individual genetic ancestry among six Fea’s muntjac individuals and one putative hybrid individual at K = 2–5. Each vertical bar represents an individual, with colors indicating inferred ancestral components and bar heights corresponding to estimated ancestry proportions. The institution of origin of each individual is indicated.

Individual genetic ancestry was further inferred using ADMIXTURE v. 1.3.0. Cross-validation analysis of the Fea’s muntjac dataset revealed that K = 3 yielded the lowest cross-validation (CV) error (Suppl. material 2). However, given the limited sample size and the known number of potential source populations, K values ranging from 2 to 5 were examined to visualize and interpret ancestry patterns (Fig. 6). At K = 2, the putative hybrid individual was clearly separated from all Fea’s muntjac individuals. At K = 3, individuals from TNC (FM1F and FM2M) and DSZ (FM5M, FM6M and FM7M) shared a predominant ancestral component, while FM4F and the putative hybrid individual formed distinct clusters. Increasing the number of clusters to *K* = 4 provided greater resolution by separating the TNC and DSZ populations, consistent with their known institution of origin. Notably, FM7M exhibited an admixed ancestry pattern, with approximately 30% of its inferred ancestry shared with the DSZ cluster, suggesting partial admixture within this group.

### Genetic relatedness estimation using IBD analysis

**Figure 7.**
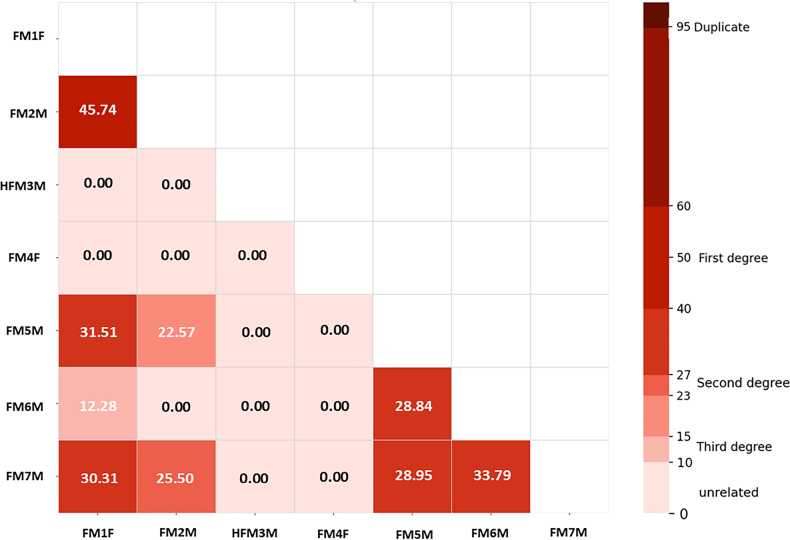
Heatmap of pairwise PI_HAT values (PI_HAT × 100) among Fea’s muntjac (FM) individuals showing estimated genetic relatedness. Darker colors indicate higher relatedness values. Approximate relatedness categories are indicated on the color scale, ranging from unrelated individuals (PI_HAT ≈ 0) to duplicate or nearly identical samples (PI_HAT × 100 > 95).

Genetic relatedness among ex-situ Fea’s muntjac individuals was assessed by estimating pairwise identity-by-descent (IBD) using 9109 filtered SNPs, with the same SNP filtering criteria applied in the phylogenetic analysis. A heatmap of pairwise PI_HAT values revealed that FM1F and FM2M were identified as first-degree relatives. FM5M, FM6M and FM7M exhibited second-degree relatedness to one another. Additional second-degree relationships were detected between FM1F and FM2M and between individuals FM5M and FM7M. In contrast, FM4F and the putative hybrid individual (HFM3M) showed no evidence of genetic relatedness to any other individuals in the dataset (Fig. 7).

## Discussion

This study provides a genome-wide assessment of relatedness and population structure among the remaining ex-situ Fea’s muntjac in Thailand. By integrating evidence from phylogenetic inference, ADMIXTURE clustering, and IBD analyses, we identified consistent genetic patterns that largely correspond with available pedigree information. These findings underscore the value of SNP-based genomic tools for validating incomplete or uncertain historical records and for informing conservation management in small, fragmented ex-situ wildlife populations, consistent with applications reported in other managed species, including Eld’s deer and scimitar-horned oryx (Ivy et al. 2016; Pumpitakkul et al. 2023). The strong genetic clustering of FM1F and FM2M reflects their documented full-sibling relationship and confirms the reliability of RADseq-derived kinship estimates in small populations. Similarly, the second-degree relatedness observed among FM5M, FM6M, and FM7M is consistent with their recorded half-sibling relationships. Together, these results demonstrate how genomic data can corroborate pedigree records and reconstruct family structure when documentation is sparse or inaccurate, a common challenge in long-established ex-situ populations (Willis 1993). Comparable applications of SNP-based kinship analysis have been reported in other managed species, including Baer’s pochard and addax, where molecular data helped resolve missing or ambiguous pedigree information (Ivy et al. 2016; Chaille et al. 2024).

Beyond corroborating known relationships, the genomic data also revealed relatedness patterns not captured in existing pedigrees. The second-degree relatedness detected between FM5M and FM6M suggests an unrecorded familial connection. Similar genome-wide SNP analyses in Eld’s deer have demonstrated clear genetic differentiation between the Siamese and Burmese subspecies maintained under the ZPOT and the DNP, and further revealed translocations of two Siamese Eld’s deer individuals between institutions (Pumpitakkul et al. 2023). In the present study, the elevated relatedness observed between individuals from TNC (FM1F and FM2M) and DSZ (FM5M and FM7M) likely reflects increased background relatedness among the founders rather than recent shared ancestry. Such patterns are commonly observed in small or bottlenecked wildlife populations, where limited founder numbers and genetic drift increase the extent of shared genomic segments across individuals (Knief et al. 2015). This explanation is plausible for Fea’s muntjac, which has experienced long-term population decline and was established from a very small number of ex-situ founders. Consistently, measures of genomic inbreeding and heterozygosity for these individuals have been previously reported and are consistent with the severely bottlenecked founder history inferred from the kinship patterns described here (Supapannachart et al. 2026). Based on the genomic findings of this study, several conservation management recommendations are proposed for the ex-situ Fea’s muntjac population in Thailand. Mating between FM1F and FM2M should be avoided, given their confirmed first-degree relatedness, and pairings within the DSZ lineage should be managed cautiously due to evidence of second-degree relationships. These findings highlight the value of coordinated breeding management and genomically informed pairing strategies for the ex-situ Fea’s muntjac population. The genetic distinctiveness of FM4F from KKOZ has important implications for conservation planning. FM4F showed no detectable IBD relationships with any other individual and consistently formed an independent lineage across all analyses, indicating the presence of unique genetic variation that could enhance overall diversity within the ex-situ population. Notably, the pronounced distinctiveness of FM4F across analyses raises the possibility of cryptic hybridization. Nevertheless, both the phylogenetic and ADMIXTURE analyses support its classification as a pure Fea’s muntjac lineage, as FM4F remained within the Fea’s muntjac clade and clustered with Fea’s muntjac individuals at K = 2. Definitive exclusion of cryptic hybridization would nonetheless require cytogenetic analysis, given the remarkable karyotypic diversity documented across muntjac species despite their morphological similarity (Fontana and Rubini 1990; Huang et al. 2006). Given the advanced age of FM4F, future preservation of somatic tissues for cryobanking and fibroblast culture may provide an important strategy for conserving its unique genetic variation (Hildebrandt et al. 2018; Herrick 2019).

The putative hybrid individual (HFM3M) formed a distinct genetic cluster across all analyses, supporting the utility of RADseq for detecting hybridization even in cases where morphological evidence alone may be ambiguous (Hohenlohe et al. 2011; Rutledge et al. 2015). Comparable RADseq-based approaches have successfully identified cryptic hybrids in other taxa, including trout and North American *Canis* species, enabling the resolution of uncertain ancestry and introgression patterns (Hohenlohe et al. 2011; Rutledge et al. 2015). In the present study, the genomic evidence for hybrid ancestry was corroborated by intermediate coat coloration and atypical fecal characteristics. HFM3M was genetically distinct from all Fea’s muntjac individuals in both phylogenetic and ADMIXTURE analyses, supporting its putative hybrid origin. Although cytogenetic analysis was not performed, future karyotypic characterization would provide definitive confirmation and additional resolution of chromosomal composition, given the remarkable karyotypic diversity documented across muntjac species (Fontana and Rubini 1990; Huang et al. 2006). Nonetheless, HFM3M should be excluded from Fea’s muntjac breeding programs to prevent potential introgression of non-Fea’s muntjac genetic material into the ex-situ population.

Several limitations of this study warrant consideration. The number of individuals analyzed reflects the critically depleted status of the ex-situ population rather than a study design choice, as the wild population of Fea’s muntjac in Thailand remains poorly characterized and has been classified as Data Deficient on the IUCN Red List since 1996, with no quantitative population estimate currently available (Timmins et al. 2016). The relatively small sample size, including the exclusion of one individual and the identification of one putative hybrid, may limit statistical power and reduce the generalizability of the inferred population structure patterns. Therefore, the findings should be interpreted with appropriate caution. Nevertheless, previous simulation and empirical RADseq studies have demonstrated that meaningful population genetic inference can still be achieved from small sample sets when sufficiently large numbers of bi-allelic SNPs are employed (Willing et al. 2012; Nazareno et al. 2017; Qu et al. 2019). Importantly, our dataset substantially exceeded these thresholds, comprising over 9000 high-quality SNPs for structure analyses and more than 67,000 LD-pruned SNPs for ADMIXTURE and IBD analyses. The findings presented here should therefore be viewed as an important genomic baseline that can be expanded as additional individuals become available through captive breeding, wildlife rescue, or confiscation efforts. Collectively, the genomic analyses revealed clear genetic structuring within the ex-situ Fea’s muntjac population in Thailand, separating individuals into three major groups: a cluster of related individuals originating from DSZ and TNC, a genetically distinct individual (FM4F) from KKOZ, and a putative hybrid. Concordant results from phylogenetic, IBD, and ADMIXTURE analyses not only validated available pedigree records but also uncovered previously unrecognized familial relationships, highlighting the limitations of pedigree-based management in extremely small ex-situ populations (Willis 1993; Ivy et al. 2016). Overall, this study demonstrates the substantial value of RADseq-based genomic assessments for conservation management, particularly in contexts where population sizes are critically small and pedigree information alone is insufficient to guide informed breeding and long-term conservation planning.

## Conclusions

This study provides a genome-wide SNP-based assessment of genetic diversity, relatedness, and population structure in the remaining ex-situ Fea’s muntjac population in Thailand. Using RADseq-derived data, we demonstrate that genomic approaches offer substantial advantages over pedigree information alone, particularly in extremely small and genetically constrained populations. The results establish an essential genomic baseline for Fea’s muntjac and provide a practical, scalable framework for integrating genomics into conservation breeding and management. This approach may also be extended to future conservation initiatives for Fea’s muntjac and other threatened cervid species maintained under human care.

## Additional information

### Artificial Intelligence (AI) use

Regarding the use of AI in the preparation of this manuscript, the authors declare the following:

AI-assisted tools were used only for language polishing and improving readability. The scientific content, data analysis, and conclusions were entirely developed by the authors.

### Author ORCIDs

P. Supapannachart https://orcid.org/0009-0005-1025-1406

S. Tangphatsornruang https://orcid.org/0000-0003-2673-0012

W. Pootakham https://orcid.org/0000-0001-6721-6453

C. Sonthirod https://orcid.org/0009-0009-3116-0078

S. Tongsima https://orcid.org/0000-0002-1491-1839

P. Wangkumhang https://orcid.org/0000-0003-1552-3149

A. Thongphakdee https://orcid.org/0000-0002-4086-7245

G. Suriyaphol https://orcid.org/0000-0002-8961-5970

### Data availability

All of the data that support the findings of this study are available in the main text or Supplementary Information.

## Supplementary materials

Supplementary material 1 Supplementary table Data type docx Explanation note RADseq sequencing output and read-mapping statistics for six Fea’s muntjac individuals and one putative hybrid aligned to the *Muntiacus feae* reference genome. https://binary.pensoft.net/file/1696690This dataset is made available under the Open Database License (http://opendatacommons.org/licenses/odbl/1.0/). The Open Database License (ODbL) is a license agreement intended to allow users to freely share, modify, and use this Dataset while maintaining this same freedom for others, provided that the original source and author(s) are credited. Pannawat Supapannachart, Sithichoke Tangphatsornruang, Wirulda Pootakham, Chutima Sonthirod, Sissades Tongsima, Pongsakorn Wangkumhang, Alisa Wilantho, Ampika Thongphakdee, Saowaphang Sanannu, Pongsatorn Kongjak, Thewarach Vechmanus, Supatsorn Panyalert, Gunnaporn Suriyaphol

Supplementary material 2 Supplementary image Data type png Explanation note Cross-validation (CV) error plot across K values ranging from 1 to 5 for six Fea’s muntjac individuals and one putative hybrid individual. https://binary.pensoft.net/file/1696691This dataset is made available under the Open Database License (http://opendatacommons.org/licenses/odbl/1.0/). The Open Database License (ODbL) is a license agreement intended to allow users to freely share, modify, and use this Dataset while maintaining this same freedom for others, provided that the original source and author(s) are credited. Pannawat Supapannachart, Sithichoke Tangphatsornruang, Wirulda Pootakham, Chutima Sonthirod, Sissades Tongsima, Pongsakorn Wangkumhang, Alisa Wilantho, Ampika Thongphakdee, Saowaphang Sanannu, Pongsatorn Kongjak, Thewarach Vechmanus, Supatsorn Panyalert, Gunnaporn Suriyaphol
