# Supplementary material for: Genome-wide SNP data reveal genetic relatedness and structure in an ex-situ population of threatened Fea's muntjac (Muntiacus feae) (Artiodactyla, Cervidae)
Source: Zookeys. 2026 Jun 26;1283:223–39. doi: 10.3897/zookeys.1283.186911 (PMC13332386; doi:10.3897/zookeys.1283.186911)
Supplement: Supplementary material 1 — Supplementary table [file zookeys-1283-223_article-186911__-s001.docx]

**Supplementary Table S1.** RADseq sequencing output and read-mapping statistics for six Fea’s muntjac individuals and one putative hybrid aligned to the *Muntiacus feae* reference genome

| **Sample ID** | **Total paired-end reads** | **Mapped reads** | **Alignment rate (%)** |
| --- | --- | --- | --- |
| **Fea’s muntjac** | | | |
| FM1F | 12,107,994 | 11,861,165 | 97.96 |
| FM2M | 9,009,642 | 8,835,388 | 98.07 |
| FM4F | 13,619,414 | 13,333,340 | 97.90 |
| FM5M | 7,950,662 | 7,710,150 | 96.97 |
| FM6M | 8,977,898 | 8,622,263 | 96.04 |
| FM7M | 20,043,634 | 19,204,006 | 95.81 |
| **Putative hybrid Fea’s muntjac** | | |  |
| HFM3M | 11,726,850 | 11,396,711 | 97.18 |
