# Supplementary figures and images for: Genome-wide SNP data reveal genetic relatedness and structure in an ex-situ population of threatened Fea's muntjac (Muntiacus feae) (Artiodactyla, Cervidae)
Source: Zookeys. 2026 Jun 26;1283:223–39. doi: 10.3897/zookeys.1283.186911 (PMC13332386; doi:10.3897/zookeys.1283.186911)

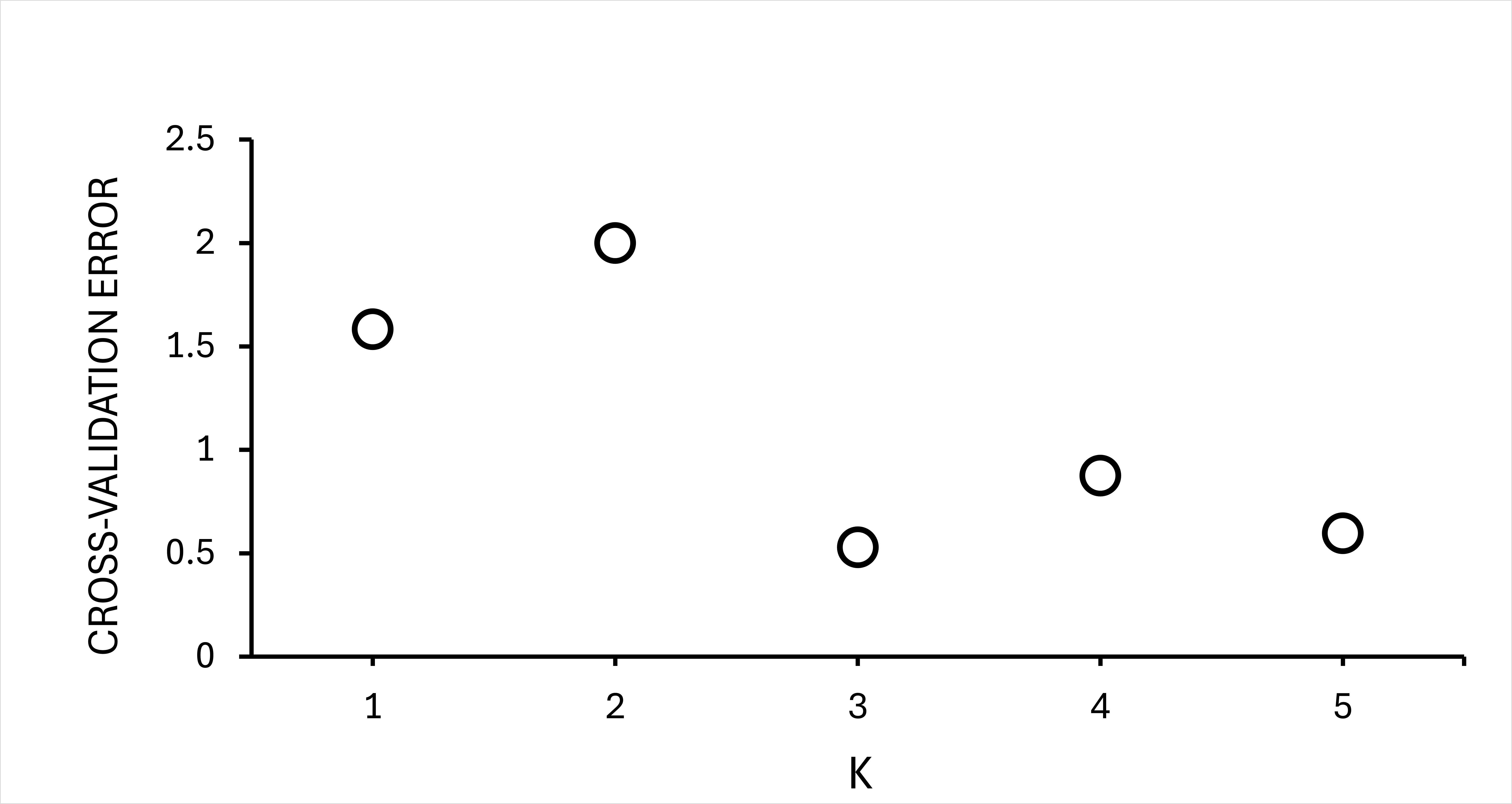

Supplement: Supplementary material 2 — Supplementary image [file zookeys-1283-223_article-186911__-s002.png]
